# A “Fork-to-Farm” Multi-Scale Approach to Promote Sustainable Food Systems for Nutrition and Health: A Perspective for the Mediterranean Region

**DOI:** 10.3389/fnut.2018.00030

**Published:** 2018-05-22

**Authors:** Eric O. Verger, Marlene Perignon, Jalila El Ati, Nicole Darmon, Marie-Claude Dop, Sophie Drogué, Sandrine Dury, Cédric Gaillard, Carole Sinfort, Marie-Josèphe Amiot, MEDINA-Study Group, Marie-Josèphe Amiot

**Affiliations:** ^1^NUTRIPASS, IRD, Univ Montpellier, SupAgro, Montpellier, France; ^2^MOISA, Univ Montpellier, CIHEAM-IAMM, CIRAD, INRA, Montpellier SupAgro, Montpellier, France; ^3^Research Laboratory SURVEN Nutritional Surveillance and Epidemiology in Tunisia, National Institute of Nutrition and Food Technology (INNTA), Tunis, Tunisia; ^4^ELSA Group, ITAP, SupAgro, Irstea, Univ Montpellier, Montpellier, France

**Keywords:** food system, diet, sustainability, conceptual framework, life cycle analysis, local agro-ecosytem, mathematical optimization, multi-criteria mapping

## Abstract

Mediterranean countries are undergoing dietary and nutritional changes that affect their inhabitants' health, while facing massive environmental challenges. The increasing demand of water in agriculture, the capacity to maintain local food production, and the growing dependence on food imports are interconnected issues that must be addressed to ensure food security and nutrition in the Mediterranean region. Here, we present the conceptual framework and methodologies developed by the MEDINA-Study Group for rethinking food systems toward sustainable consumption and production modes. Based on its multidisciplinary expertise, the MEDINA-Study Group designed a “fork-to-farm” multi-scale approach, stemming from current dietary habits and examining how some options to nutritionally improve these habits might affect the food systems. This approach was developed for research activities in the South of France and Tunisia, two areas with very different diet-agriculture-environment nexus. The conceptual framework is based on the analysis of elements of the food systems (from consumption to production) at different levels (individual, household, regional and national levels). The methods include: (i) modeling options of dietary changes at different scales, in order to nutritionally optimize food consumption-production without increasing the environmental impact, (ii) translating the best-choice changes into possible policy actions, (iii) testing the acceptability and feasibility of these actions with several stakeholders, and (iv) producing guidelines for sustainable food choices and production. The MEDINA-Study Group identified additional issues that could be included in a future framework to help designing ambitious agricultural, food and health policies in the Mediterranean region.

## Introduction

Food systems have long been described as chains of activities from food production (“the farm”) to consumption (“the fork”), including processing, packaging, distribution and retail. These activities are influenced by many political, economic, socio-cultural, and environmental factors (1). Indeed, recently, food systems have been conceptualized as complex socio-ecological systems that involve cross-level and cross-scale interactions between human and natural components and major social outcomes, such as ecosystem services, social welfare and food security (2). Food security is a situation that exists when all people, at all times, have physical, social and economic access to sufficient, safe and nutritious food that meets their dietary needs and food preferences for an active and healthy life (3).

The 2015 Food and Agriculture Organization (FAO) report on the State of Food Insecurity in the World considered that most of the Southern Europe, Middle East and North Africa countries that constitute the Mediterranean region have eradicated severe food insecurity (4). Nevertheless, the traditional Mediterranean dietary pattern has progressively declined in the last 50 years (5–7). Concomitantly, food habits in the Mediterranean countries have largely evolved with more consumption of animal-based products and ultra-processed foods, and less plant-based products, indicating a westernization of dietary patterns (8, 9). Populations exposed to this dietary shift are less likely to achieve adequate intakes of nutrients compared with people with greater adherence to the Mediterranean diet (10). Consequently, they are at high risk of obesity (11–13) and of other diet-related non-communicable diseases (14, 15). Among the whole range of determinants that have contributed to the current situation, a majority relies on the great availability and accessibility of foods typical of the Western lifestyle (highly processed, calorie-rich, nutrient-poor food and animal products). This has been facilitated by activities of transnational food corporations (e.g., food marketing and retailing systems), trade liberalization policies, and sometimes government subsidies (16).

Simultaneous to the dietary and nutritional shift, the Mediterranean region is facing massive environmental changes: land use and degradation, water scarcity, environment pollution, biodiversity loss, and climate change (17). Furthermore, recent analyses show that the current Western-style diet has a high environmental impact (18). While the Mediterranean region has been a major food-producing area with a large agro-biodiversity for millennia, environmental alterations may threaten the local food system capacities to ensure food and nutrition security (19).

Therefore, it is crucial to develop the concept and use of sustainable diets in different contexts (industrialized and transition countries) to ensure food security and quality (20, 21), especially in the Mediterranean region. In contrast to the “farm-to-fork” approach (from production to consumption) that is commonly used to improve the food systems in terms of food safety (22) and nutrition (23), the MEDINA research project “Promoting sustainable food systems in the Mediterranean for good nutrition and health” proposes a “fork-to-farm” conceptual framework. Stemming from the current dietary habits of the Mediterranean population, this program aims to identify the dietary changes needed to ensure quantitative and qualitative food security, while examining how these changes might affect the whole food systems.

The first part of this article presents the relevant parameters to be included in this conceptual framework for rethinking food systems in order to promote sustainable consumption and production in the Mediterranean region. Then, it describes the approach and the methodologies developed by the MEDINA-Study Group to identify the dietary changes needed (i) to nutritionally optimize food consumption-production without increasing the environmental impact, and (ii) to assess the acceptability and feasibility of these changes by the various food system stakeholders. The last part presents and discusses the perspectives for implementing other relevant parameters in the initial framework.

## Parameters affecting the sustainability of food systems in the mediterranean region

The MEDINA-Study Group includes experts in nutrition, food science, epidemiology, biostatistics, agronomy, environmental science and economics. Based on literature review and discussions, the MEDINA-Study Group identified relevant parameters to be included in a conceptual framework to assess the sustainability of food systems in the South of France and Tunisia, two completely different areas concerning the diet-agriculture-environment nexus.

### Adherence to the mediterranean diet pyramid

The Mediterranean basin is the cradle of the “traditional Mediterranean diet” that is proposed as a healthy diet model to prevent coronary heart diseases (24), and has been registered by UNESCO in 2010 as an intangible heritage of humanity (25). Prospective and interventional studies in the Mediterranean region confirmed that good adherence to this dietary pattern is systematically associated with a markedly reduced risk of obesity (26), metabolic syndrome (27–29), type 2 diabetes (30, 31), and cardiovascular diseases (32, 33).

In addition, Mediterranean traditional foods contain a large diversity and quantity of micronutrients and bio-active compounds (34) and a way to meet the nutrient requirements (10). Interestingly, computer-generated personalized diets emphasize the need to increase the consumption of foods typical of the Mediterranean diet (nuts, unrefined grains, vegetables and legumes, fruit, fish, and shellfish) to fulfill the individual nutrient requirements (35). In the Mediterranean region, promoting the adherence to the Mediterranean diet pyramid (36) is relevant for ensuring good health and nutrition.

Moreover, the Mediterranean diet pyramid takes into account the local and seasonal declination of this dietary pattern around the Mediterranean border (5), as well as the associated know-how, knowledge, practices and traditions (37). Therefore, it is important also to assess the nutritional value and nutrient profile of local products and recipes (38).

To ensure good health and nutrition while respecting the socio-cultural context of the Mediterranean region, the adherence to the Mediterranean diet pyramid will be included in the conceptual framework as an objective of the optimization mathematical models (see section The MEDINA “Fork-to-Farm” Multi-Scale Approach).

### Nutritional potential of local agro-ecosystems

In addition to the nutritional value and nutrient profile of local products and recipes, it is also important to understand the nutritional potential of local agro-ecosystems, including wild edible plants and animals that are currently neglected and underused. The potential role of domesticated and wild biodiversity in addressing food security, nutrition deficiencies and the emerging burden of non-communicable diseases was emphasized by the cross-cutting initiative on biodiversity for food and nutrition (39). Since then, it has been acknowledged that the proper management of the local domesticated and wild biodiversity significantly contributes to sustainable food systems by ensuring both human and environmental health (40). Furthermore, understanding the nutritional potential of local agro-ecosystems is a critical step when assessing how the agricultural production could contribute to the food and nutrition security inside households involved in agricultural activities. Indeed, the contribution to nutrition can be direct via self-consumption, in-kind donations and the presence of wild plants, or indirect through farm and off-farm incomes (41, 42).

The “nutritional potential of local agro-ecosystems” parameter will be included in the conceptual framework thanks to specific survey on agriculture and wild biodiversity in a rural region of Tunisia (see section The MEDINA “Fork-To-Farm” Multi-Scale Approach).

### Environmental impacts of the food systems

Many studies on the evaluation of the environmental impact of food systems use the Life Cycle Analysis (LCA) approach, a normalized method to assess all environmental impacts (43). Within the food chain, the production phase (mainly meat and dairy) has the highest impact, together with transport, packaging and food loss/waste (44). The main contributors are greenhouse gas (GHG) emissions, water deprivation, and land use for production (transformation and occupation). This causes biodiversity loss and affects soil quality, particularly the use of toxic products, mainly pesticides, in farmed soil (44).

It has been acknowledged that a better adherence to the Mediterranean diet reduces the environmental impact thanks to the consumption of more plant-derived products and fewer animal products, whereas a Western-style diet is associated with high environmental impacts (45–47). Furthermore, due to the increasing complexity of the food chain, there is a trend to have a greater flow of food commodities over long distances, and to consume highly processed and packaged foods that contribute to increase GHG emissions and deplete non-renewable resources (48). Finally, as the Mediterranean population represents 60% of the population of water-scarce countries (48), particular attention should be paid to water use.

To ensure the sustainability of the food systems in the Mediterranean region, the “environmental impacts of the food systems” parameter will be included in the conceptual framework as constraints of the optimization mathematical models (see section The MEDINA “Fork-To-Farm” Multi-Scale Approach).

### Food trade and dependence on food imports

Since 1965, the importance of food trade has increased worldwide, and progressively more people are becoming dependent on food imports to ensure food availability (49). In the Mediterranean basin, France is a strong agro-food exporting country, whereas other Mediterranean countries are more and more dependent on food imports (49). Indeed, except for France, all Northern Mediterranean countries (e.g., Italy and Spain) suffered from a dramatic decline in food self-sufficiency (49).

To take into account the importance of the “food trade and dependence on food imports” parameter, the conceptual framework will include national food availability data (FAO food balance sheets) which provide information of food production but also imports and exports (see section The MEDINA “Fork-To-Farm” Multi-Scale Approach).

## The MEDINA “fork-to-farm” multi-scale approach

The aim of the MEDINA research project is to propose guidelines and policy actions to promote sustainable food systems for ensuring good health and nutrition for the Mediterranean populations, while mitigating the environmental impact and promoting the local cultural heritage and traditional food products.

**Figure 1:**
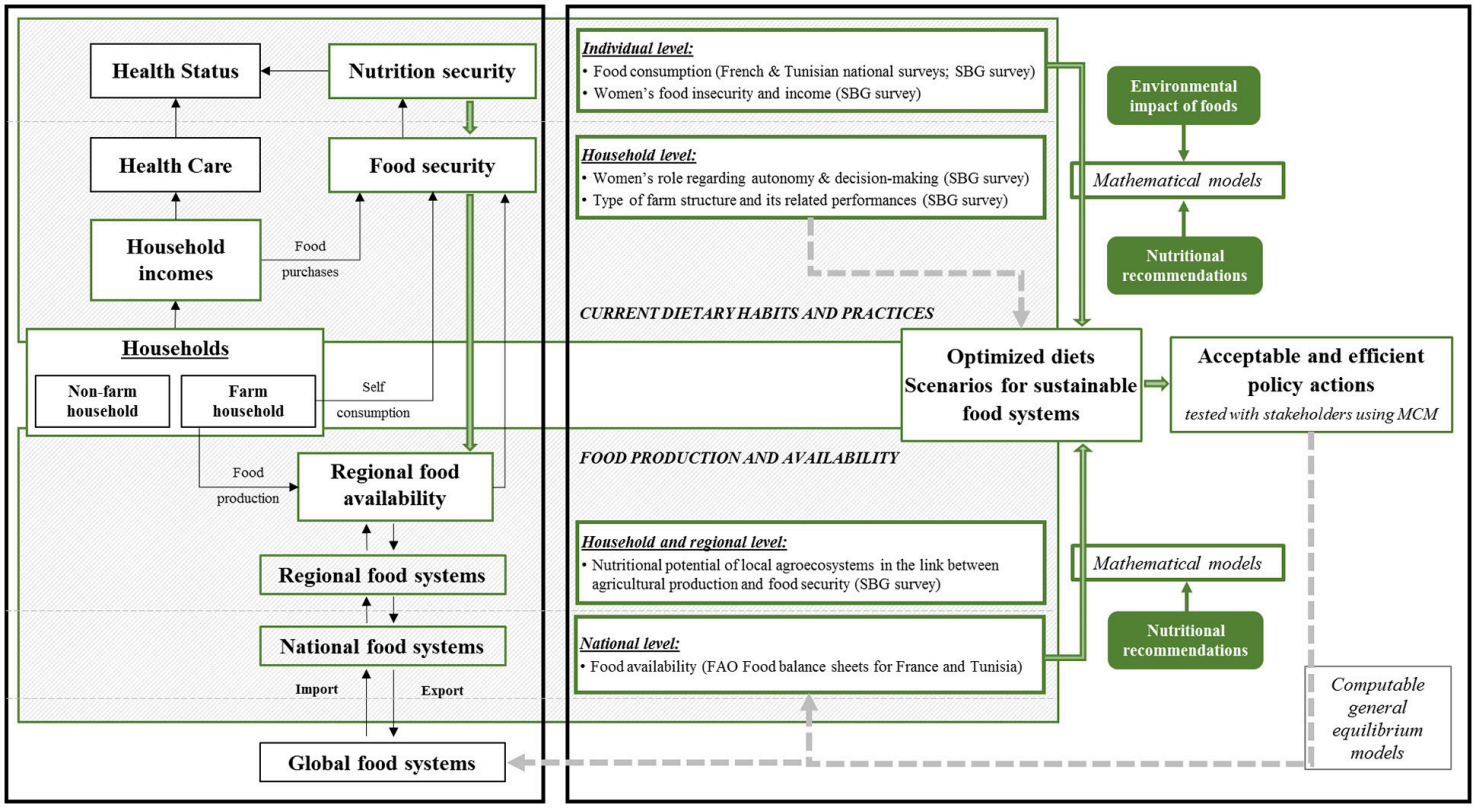
Conceptual framework developed by the MEDINA-Study Group to provide solid support for revised guidelines compatible with sustainable food systems that promote good health and nutrition and also the local cultural heritage and traditional food products. The left side of the figure briefly summarizes how food systems, nutrition and health are interconnected and affect each other in the classical “farm-to-fork” approach (black arrows) and the “fork-to-farm” approach used in the MEDINA research project (green arrows). The right side represents the modeling approach used to propose guidelines to ensure good nutrition and sustainability, with the models currently implemented in the MEDINA research project (green arrows and boxes) and the models considered as research perspectives (gray arrows and boxes). Both sides consider different scales (individual, household, regional and national levels) by using different databases. SBG, Sidi Bouzid governorate; MCM, Multi-Criteria Mapper computer program.

The first step of the “fork-to-farm” multi-scale approach developed by the MEDINA-Study Group (Figure 1) is to determine the current, individual and household dietary habits and practices. This information will be used to identify the dietary changes needed to ensure food and nutrition security. The second step is to evaluate how these changes could affect food production and availability at the household, regional and national scales.

Analysis at different scales are possible by using existing datasets and by performing new surveys specifically for the MEDINA project (Figure 1). Existing food consumption datasets representative of the adult population living in the South of France (50) and Tunisia (TAHINA: Epidemiological Transition and Health Impact in North Africa—Contract Number: ICA3-CT-2002-10011) will be used to define the current dietary habits and practices (individual level). A specific survey on the women's individual food intake and nutrition was carried out to determine the current dietary habits and practices in relation to agriculture and wild biodiversity in the Sidi Bouzid governorate (individual level). This Tunisian governorate is home to a population whom 73% is settled in a rural area and where 46% of active women are employed in the agriculture (51), a context which strongly differs with South of France where agriculture concerned less than 2% of active women (52). A budget survey focused on women, their household and, where appropriate, on the farm where they live will be organized in this governorate to assess the current dietary habits and practices, farm production, and food availability (individual and household levels). This multi-level survey will allow (i) determining the farm structure types and performances, (ii) understanding the women's place and role within the farm and the household concerning autonomy and decision-making, (iii) assessing the determinants of women's food insecurity relative to their income in urban and rural situations, and (iv) integrating the nutritional potential of local agro-ecosystems in the link between agricultural production and food security within farm households. Existing data on food availability (FAO food balance sheets) for the general French and Tunisian populations will be used to define the food production and availability (national level).

To identify the dietary changes needed to reach nutritional adequacy and how these changes might affect the whole food systems, two specific models will be applied using different datasets (Figure 1). The first model will be based on the current dietary habits and practices (individual level). Specifically, the mean diet observed in the studied populations will be estimated from individual food consumption surveys. Then, mathematical models will be used to design optimized diets that fulfill the nutritional recommendations and/or the Mediterranean pyramid guidelines, while minimizing the departure from the mean diet in order to suggest realistic dietary changes. The environmental impacts (water deprivation, land use effects including biodiversity) of these optimized diets will be assessed to evaluate the compatibility between the nutritional and environmental dimensions of sustainability. More stringent models that integrate environmental constraints will be developed to design diets that fulfill the nutritional recommendations without increasing the environmental impacts (53–55).

The second model will be based on food production and availability (national level). Specifically, based on the quantities of food available for human consumption (estimated using the FAO food balance sheets), a mathematical model will be used to optimize food availability relative to nutrient requirements, while minimizing the departure from the mean diet. Then, the impacts of these changes on the food chains and general economy will be evaluated.

The different surveys and models used in the MEDINA project will provide specific outputs, such as optimized diets and scenarios for sustainable food systems. Based on these outputs, guidelines and policy actions to promote sustainable food systems for ensuring health and nutrition for the Mediterranean populations could be designed.

One way to ensure the efficiency of the future guidelines is to involve key stakeholders during the development process. This will be done through three steps: (i) translation of the previously identified sustainable dietary changes into a range of different policy actions based on discussions within the MEDINA-Study Group; (ii) collecting, via structured interviews, the opinion of key stakeholders on the overall performance of the pre-defined policy actions. This will be done using a multi-criteria mapping technique based on the “Multi-Criteria Mapper” computer program (56); and (iii) providing useful guidelines to policy-makers on sustainable food choices and production based on the policy actions that were most valued by the participant stakeholders.

Furthermore, the MEDINA framework includes analysis and models considered as research perspectives. A first perspective is the use of a spatialized analysis to assess the impact of water deprivation and land use occupation and transformation in other countries linked to the import of food (or animal feed) products. Another perspective is the use of a multi-sectoral, multi-regional computable general equilibrium (CGE) model allow to consider the complexity of the relations that link countries in a global economy, as well as the possible feedback associated with exogenous shocks.

## Preliminary results obtained from the MEDINA framework

The MEDINA “fork-to-farm” is a promising framework for rethinking food systems toward sustainable consumption and production modes thanks to its multi-scale approach, as illustrated by the following preliminary results obtained at individual level and national level for Tunisia.

Using the first model based on the current dietary habits and practices (individual level), we found that the main dietary changes needed to satisfy all the nutrient recommendations were the increases of fruits and dairy products, and decreases of meat and starchy foods. Nevertheless, these changes increased the environmental impacts of the diets. In a scenario where environmental indicators were limited to their observed levels, the dietary changes needed were still the decreases of meat and starchy foods but also lower increases of fruits and dairy products in favor of vegetables (57).

Using the second model based on food production and availability (national level), we found that the main changes in food availability needed to satisfy all the nutrient recommendations—without considering the environmental impacts—were the reduction of the imports of starchy foods, sugar, meat, soybean and palm oil and a moderate reduction of olive oil exports (58).

## Recommendations of future research and actions

Although the “fork-to-farm” multi-scale approach will be useful for designing relevant agricultural, food and health policies, the MEDINA-Study Group identified other factors to improve the framework.

Adhering to the Mediterranean diet pyramid seems to be relevant for ensuring good health and nutrition; however, the diet nutritional adequacy depends on the total nutrients intake and bioavailability, defined as the proportion of an ingested nutrient that is absorbed and utilized through normal metabolic pathways (59). When promoting more sustainable diets that imply a shifts from animal-based toward plant-based products, changes in nutrient bioavailability, which strongly depends on the food source (animal vs. plant) and diet composition (e.g., iron, zinc, protein, and vitamin A), should be assessed and integrated in the future design of optimized diets (60).

Another relevant aspect for ensuring good health and nutrition concerns the assessment of the chemicals used in agriculture to increase the efficiency and yield (fertilizers, pesticides, hormones, antibiotics). The general population could be exposed to pesticides by ingestion of food and drinking water. Moreover, specific populations, such as farmers and farm workers, could also be directly exposed by dermal contact or inhalation (61, 62). Many studies have documented how pesticide exposure can negatively affect health, being related to various diseases including cancers, leukemia and asthma (52). Future research should address this point, with a holistic methodology that takes into account the exposures to dietary contaminants when respecting nutrient recommendations (63), but also production efficiency and yield.

In their modeling study, Tilman et al. stressed the importance of assessing the impact of consumption changes on the environment, particularly GHG emissions, water deprivation and land use (land clearing and habitat fragmentation, soil quality 64). Agriculture has an important role to play in reducing GHG emissions, decreasing fossil energy consumption, optimizing the nitrogen life cycle to limit nitrogen inputs, enhancing soil carbon sequestration, and improving the use of manure and slurry. Future research should adopt a global comprehensive approach to evaluate the sustainability of these trade balances which is necessary for the survival of agriculture in most countries at risk (65).

Behind the “fork-to-farm” approach there is also the idea that in a globalized economy, aggregated individual decisions affect also the global economy. This is the case when dietary habits change at the country level. To assess the possible outcome of dietary recommendations, we need models that can reproduce the relationships between domestic demand and supply in the agricultural sector, and between agriculture and the rest of the economy. These models must also summarize the links between a country and the rest of the world through trade. Future research should integrate CGE models like the Global Trade Analysis Project (GTAP) model maintained by the University of Purdue which allows simulating the scenarios of change (66), built from the results of the optimization models at the individual and aggregated levels. The GTAP approach allows going back-and-forth between various scale levels.

While the specific optimization models used in the MEDINA project focus on the individual and national levels, future work should concern the regional level. Moreover, if the resulting scenarios provide the basis for actions and discussions with policy-makers and agro-food chain stakeholders, the next step will be to work on the pathways from current consumption, agricultural production and agro-food chain organization toward the future sustainable scenarios.

## Conclusion

To promote sustainable food systems for nutrition and health in the Mediterranean region, the MEDINA-Study Group developed a conceptual framework and methodologies for rethinking food systems. The group also identified additional issues that can be included in the initial framework, such as nutrient bioavailability and exposure to contaminants and active substances used in agriculture, and that could contribute to the design of ambitious agricultural, food and health policies and to action prioritization.

## Author contributions

EV, MP, and M-JA wrote the first draft of the manuscript; JE, ND, M-CD, SoD, SaD, CG, and CS contributed to writing the manuscript and offered critical comments; and all authors read and approved the final manuscript.

### Conflict of interest statement

The authors declare that the research was conducted in the absence of any commercial or financial relationships that could be construed as a potential conflict of interest. The reviewer RL and handling Editor declared their shared affiliation.
